# Relationship Between Percutaneous Transhepatic Cholangioscopy Findings and Pattern of Carcinomatous Spread in the Bile Duct

**DOI:** 10.1155/DTE.1.45

**Published:** 1994

**Authors:** M. Sato, I. Maetani, S. Ohashi, S. Ogawa, T. Anzai, H. Hoshi, H. Yoshioka, Y. Igarashi, Y. Sakai

**Affiliations:** 1 Third Department of lnternal Medicine Ohashi Hospital Toho University School of Medicine 2-17-6, Meguro-ku Tokyo 153 Japan; 2 Division of Digestive Endoscopy Ohashi Hospital Toho University School of Medicine 2-17-6, Meguro-ku Tokyo 153 Japan

## Abstract

To clarify the relationship between percutaneous transhepatic cholangioscopic findings such as papillogranular surface and vascular dilation, which are reportedly characteristic of carcinoma, and the pattern of spread for bile duct carcinomas, we compared endoscopic photographs with histological features of biopsy specimens in 57 regions of specimens from 35 patients with malignant stenosis of the bile duct. Regions with a papillogranular surface were associated with noninvasive mucosal carcinomas and papillary proliferation of superficial epithelia significantly more often than regions without such a surface (P<0.0001). The sensitivity and specificity of the papillogranular surface to noninvasive mucosal carcinoma was 79 and 95%, respectively, that of papillary proliferation of superficial epithelia was 100 and 98%, respectively. Regions with vascular dilation were associated with invasive carcinoma significantly more often than regions without vascular dilation (P<0.0001). The sensitivity and specificity of vascular dilation to invasive carcinoma were 90 and 86%, respectively. Results indicate that a papillogranular surface is related to noninvasive mucosal carcinomas while vascular dilation is related to invasive carcinomas. However, a papillogranular surface was even more closely related to papillary proliferation of superficial epithelia.


*Diagnostic and Therapeutic Endoscopy*, Vol. 1, pp. 45–50
Reprints available directly from the publisher
Photocopying permitted by license only

© 1994 Harwood Academic Publishers GmbH
Printed in Malaysia

### Relationship Between Percutaneous Transhepatic Cholangioscopy Findings and Pattern of Carcinomatous Spread in the Bile Duct

M. SATO[1], I. MAETANI[1], S. OHASHI[1], S. OGAWA[1], T. ANZAI[1], H. HOSHI[1], H. YOSHIOKA[1], Y. IGARASHI[2] and Y. SAKAI[2]

*[1]Third Department of Internal Medicine, [2]Division of Digestive Endoscopy, Ohashi Hospital, Toho University School of Medicine, 2-17-6, Meguro-ku, Tokyo, 153, Japan*

*(Received October 7, 1993; in final form December 27, 1993)*

To clarify the relationship between percutaneous transhepatic cholangioscopic findings such as papillogranular surface and vascular dilation, which are reportedly characteristic of carcinoma, and the pattern of spread for bile duct carcinomas, we compared endoscopic photographs with histological features of biopsy specimens in 57 regions of specimens from 35 patients with malignant stenosis of the bile duct. Regions with a papillogranular surface were associated with noninvasive mucosal carcinomas and papillary proliferation of superficial epithelia significantly more often than regions without such a surface ($P<0.0001$). The sensitivity and specificity of the papillogranular surface to noninvasive mucosal carcinoma was 79 and 95%, respectively, that of papillary proliferation of superficial epithelia was 100 and 98%, respectively. Regions with vascular dilation were associated with invasive carcinoma significantly more often than regions without vascular dilation ($P<0.0001$). The sensitivity and specificity of vascular dilation to invasive carcinoma were 90 and 86%, respectively. Results indicate that a papillogranular surface is related to noninvasive mucosal carcinomas while vascular dilation is related to invasive carcinomas. However, a papillogranular surface was even more closely related to papillary proliferation of superficial epithelia.

**KEY WORDS:** invasive carcinoma, malignant bile duct stenosis, noninvasive mucosal carcinoma, percutaneous transhepatic cholangioscopy

### INTRODUCTION

Since 1987, we have performed percutaneous transhepatic cholangioscopy (PTCS) with physiological saline solution after dilation of the sinus tract of percutaneous transhepatic cholangiodrainage (PTCD). PTCS is useful for diagnosing carcinomas of the pancreatobiliary system and the findings of a papillogranular surface and vascular dilation in the bile duct on PTCS reportedly indicate the presence of carcinomas (Kamiya *et al.*, 1988, 1989; Yamase *et al.*, 1988). To correctly diagnose carcinomas by PTCS, it is important to clarify the relationship between PTCS findings and the pattern of carcinoma spreads, i.e., noninvasive or invasive. To study this problem, we compared endoscopic photographs of bile ducts with the histological features of biopsy specimens.

### MATERIALS AND METHODS

In this retrospective study, we selected from our PTCS files (1987–1992) data on 35 patients with malignant bile duct stenosis for whom there existed hematoxylin and eosin (HE)-stained sections of biopsy materials containing superficial epithelia and subepithelial tissues, and good quality endoscopic photographs (Table 1).

When we performed PTCD, we dilated the sinus tract and placed a 16-Fr catheter. About 7 to 10 days later, PTCS was performed with physiological saline solution using a CHF-P20 or CHF-P10 (Olympus, Tokyo, Japan) and the biopsy materials were taken by a forcep with a needle of

Address for correspondence: Masahiro Sato, Third Department of Internal Medicine, Ohashi Hospital, Toho University School of Medicine, 2-17-6 Ohashi Meguro-ku, Tokyo 153, Japan.

**Table 1** Materials Studied

| Diagnosis | No. Patients | Regions Stenotic | Non-stenotic | Total |
|---|---|---|---|---|
| Pancreatic ca. | 15(6) | 15 | 9 | 24 |
| Bile duct ca. | 12(5) | 10 | 10 | 20 |
| Ca. of ampullary region | 3(3) | 4 | 2 | 6 |
| Ca. of gallbladder | 5(1) | 4 | 3 | 7 |
| Total | 35(15) | 33 | 24 | 57 |

(): Number of patients diagnosed by surgical resections or autopsy.

**Table 2** Histological Features of Biopsy Materials from Regions Classified by PTCS Findings

| | NIMCA(+) INVCA(+) | NIMCA(+) INVCA(−) | NIMCA(−) INVCA(+) | NIMCA(−) INVCA(−) |
|---|---|---|---|---|
| pg(+)-v(+) | | | | |
| Stenotic | 5(5)* | | | |
| Nonstenotic | 2(2) | | | |
| pg(+)-v(−) | | | | |
| Stenotic | | 2(2) | | 1(1) |
| Nonstenotic | | 2(1) | | 1(1) |
| pg(−)-v(+) | | | | |
| Stenotic | 1(0) | | 16(0) | 4(0) |
| Nonstenotic | 1(0) | | 1(0) | |
| pg(−)-v(−) | | | | |
| Stenotic | | | 2(0) | 2(0) |
| Nonstenotic | | 1(0) | 1(0) | 15(0) |

pg: papillogranular surface; v: vascular dilation; NIMCA: noninvasive mucosal carcinoma; INVCA: invasive carcinoma.

*: total number of regions and number of regions histologically showing papillary proliferation in a parenthesis.

2mm diameter. Thirty patients were examined with methylene blue staining after regular observation. The method of methylene blue staining was followed a method reported by Nishikawa *et al.* (Nishikawa *et al.*, 1991).

We classified the 57 regions in the biopsy materials from these 35 patients into 8 types, depending on whether there were stenotic regions and whether two endoscopic examinations revealed a papillogranular surface and vascular dilation, respectively, in the endoscopic photographs. The papillogranular surface was characterized as having a fluffy, granular, or papillary appearance composed of various-sized nodules or salmon roe like papillae. Vascular dilation was characterized by a proliferation and meandering of small vessels. Regions that were of the same type in the same case were counted as one. In each type of region, the pattern of carcinomatous spread and the histological structures of epithelia were examined in HE-stained sections of biopsy materials.

Eighty-two biopsy materials were available for the present study. The diagnosis of carcinoma was based on a text book of Armed Forces Institute of Pathology (Albores-Saavedra *et al.* 1986). Carcinomas were examined and categorized as either noninvasive mucosal or invasive. The biopsy materials included 19 specimens of both noninvasive mucosal carcinoma and invasive carcinoma, 6 specimens of noninvasive mucosal carcinoma, and 23 specimens of invasive carcinoma. Invasive carcinoma was differentiated from noninvasive mucosal carcinoma based on glandular structures in subepithelial tissues, according to the presence of a desmoplastic reaction surrounding the carcinoma tubules. Poorly or moderately differentiated adenocarcinoma was regarded as invasive. Superficial epithelia were examined for histological evidence of papillary proliferation.

Data are reported as number of regions. The chi-square test or Fisher's exact probability test was utilized for statistical analyses. A P value $<0.05$ was considered statistically significant.

### RESULTS

The relationship between PTCS findings and the pattern of carcinomatous spread and the histological structure of the epithelia is shown in Table 2.

### Relationship Between PTCS Findings and Pattern of Carcinomatous Spread

In all regions (7/7) with a papillogranular surface and vascular dilation, biopsy materials included both noninvasive mucosal carcinomas and invasive carcinomas (Fig. 1). In 67% (4/6) of regions with a papillogranular surface without vascular dilation, biopsy materials included noninvasive mucosal carcinomas, but no invasive carcinomas (Fig. 2); in the other two regions, biopsy materials included no carcinomas. In 83% (19/23) of the regions with vascular dilation without a papillogranular surface, biopsy materials included invasive carcinomas (Fig. 3). However, only 9% (2/23) of those specimens included noninvasive mucosal carcinomas, and 17% (4/23) included no carcinomas. In 81% (17/21) of the regions without a papillogranular surface or vascular dilation, biopsy materials included no carcinomas. Biopsy materials included only noninvasive mucosal carcinoma in one of the other regions and only invasive carcinomas in 3 of them.

There were no large differences between biopsy materials taken from stenotic regions and those from nonstenotic regions in regions with a papillogranular surface and vascular dilation, regions with a papillogranular surface without vascular dilation and regions with vascular dilation without a papillogranular surface. However, in regions without a papillogranular surface or vascular dila-

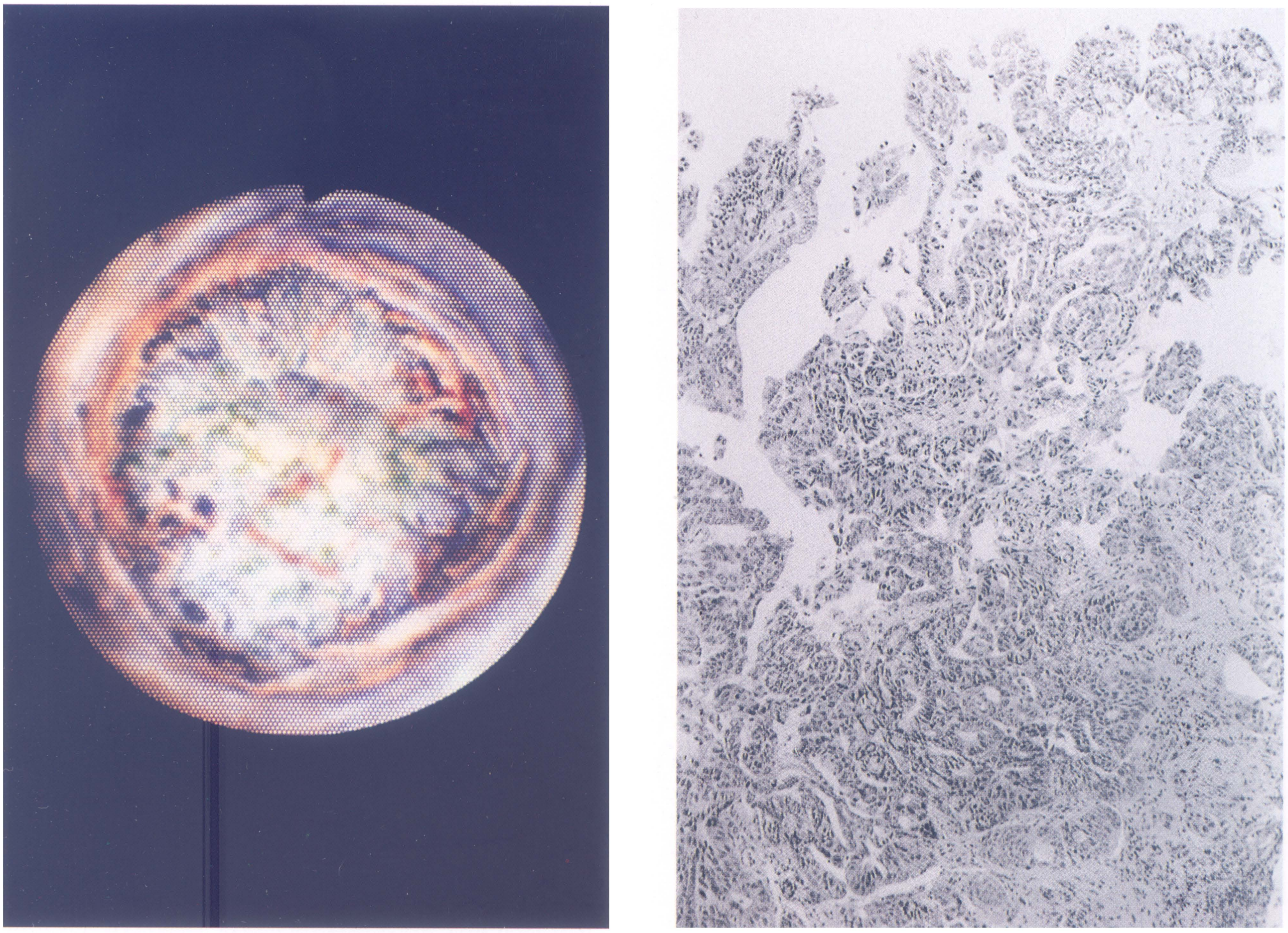

**Figure 1** A case of bile duct carcinoma. PTCS feature after dying with methylene-blue solution (left). There was a papillogranular surface and vascular dilation. Histological feature of biopsy specimen (right). Noninvasive mucosal carcinoma covered the surface of the bile duct proliferating via papillae and invasive carcinoma invaded the stroma of the bile duct wall (H-E, x25).

tion, 50% (2/4) of biopsy materials taken from stenotic regions included carcinomas, while 12% (2/17) of them from nonstenotic regions included carcinoma.

### Relationship Between PTCS Findings and Histological Structures of Epithelia

The superficial epithelia showed papillary proliferation in 12 of the 13 regions (92.3%) with a papillogranular surface. In contrast, superficial epithelia were smooth in all 44 regions without a papillogranular surface.

### Statistical Findings

Regions with a papillogranular surface were associated with noninvasive mucosal carcinomas and the papillary proliferation of superficial epithelia significantly more often than were regions without such a surface ($P<0.0001$, Table 3). The sensitivity and specificity of a papillogranular surface to noninvasive mucosal carcinoma were 79 and 95%, respectively, and those to papillary proliferation of superficial epithelia were 100 and 98%, respectively. These value for sensitivity and specificity to papillary structure of superficial epithelia exceeded those to noninvasive mucosal carcinoma. In contrast, regions with vascular dilation were associated with invasive carcinoma significantly more often than were regions without vascular dilation ($P<0.0001$). The sensitivity and specificity of vascular dilation to invasive carcinoma were 90 and 86%, respectively.

### DISCUSSION

Takada *et al.* (Takeda *et al.*, 1974) were the first to perform PTCS with a bronchofiberscopy. Since then, PTCS has been widely used for diagnosing the intracholedochal spread of carcinoma and the histological diagnosis of malignancies of the pancreatobiliary system. Many authors reported a relationship between PTCS findings and the

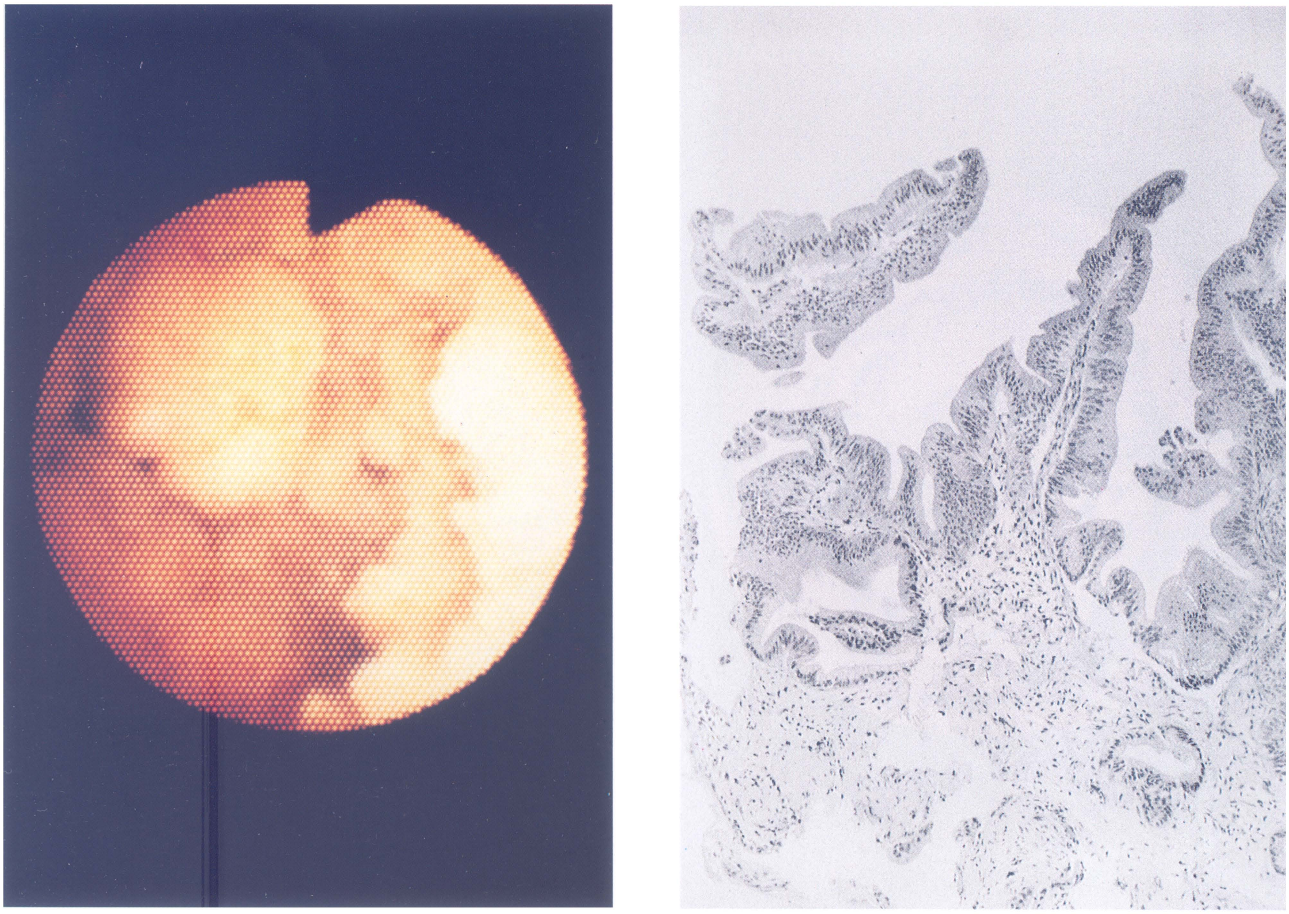

**Figure 2** A case of bile duct carcinoma. PTCS feature (left). There was a papillogranular surface but no vascular dilation. Histological feature of biopsy specimen (right). Noninvasive mucosal carcinoma covered the surface of the bile duct but no invasive carcinoma was included (H-E, x25).

rates of carcinoma positivity on biopsies (Yamase *et al.*, 1988, Nishikawa *et al.*, 1991, Sato M *et al.*, 1992) as well as characteristic PTCS findings of carcinoma of the choledochus, pancreas, and ampullary region (Yamase *et al.*, 1988, Nishikawa *et al.*, 1991). However, few investigators investigated the relationship between PTCS findings and the pattern of carcinomatous spread in detail. We therefore examined the relationship between the histological pattern of carcinomatous spread and two PTCS findings; i.e., a papillogranular surface and vascular dilation, which are characteristic of carcinomas observed on PTCS (Kamiya *et al.*, 1988, 1989; Yamase *et al.*, 1988).

A papillogranular surface of the mucosa is synonymous with a granular and papillary pattern (Kamiya *et al.*, 1988), proliferative sign (Nakazawa *et al.*, 1985) and intrabiliary protrusion (Yamase *et al.*, 1988), all described previously. Yamase *et al.* (Yamase *et al.*, 1988) reported that carcinomas were positive for papillogranular surface in endoscopic biopsies in all instances of bile duct carcinoma, carcinoma of the ampullary region, and in 75% of pancreatic carcinomas with intrabiliary protrusions. In this study, regions with a papillogranular surface were associated with noninvasive mucosal carcinomas significantly more often than were regions without such a surface. The sensitivity and specificity of a papillogranular surface to noninvasive mucosal carcinoma was 79 and 95%, respectively. These results indicate that the papillogranular surface was closely related to noninvasive mucosal carcinoma. The superficial epithelia of biopsy specimens taken from the regions with a papillogranular surface showed papillary proliferation significantly more often than did those without a papillogranular surface; the sensitivity and specificity of papillogranular surface to papillary proliferation of superficial epithelia were 100 and 98%, respectively. These sensitivity and specificity values were higher than those for noninvasive mucosal carcinoma. Therefore, a papillogranular surface was related to papillary proliferation of superficial epithelia more closely than to histological atypia. In fact, two regions associated with noninvasive mucosal carcinoma without

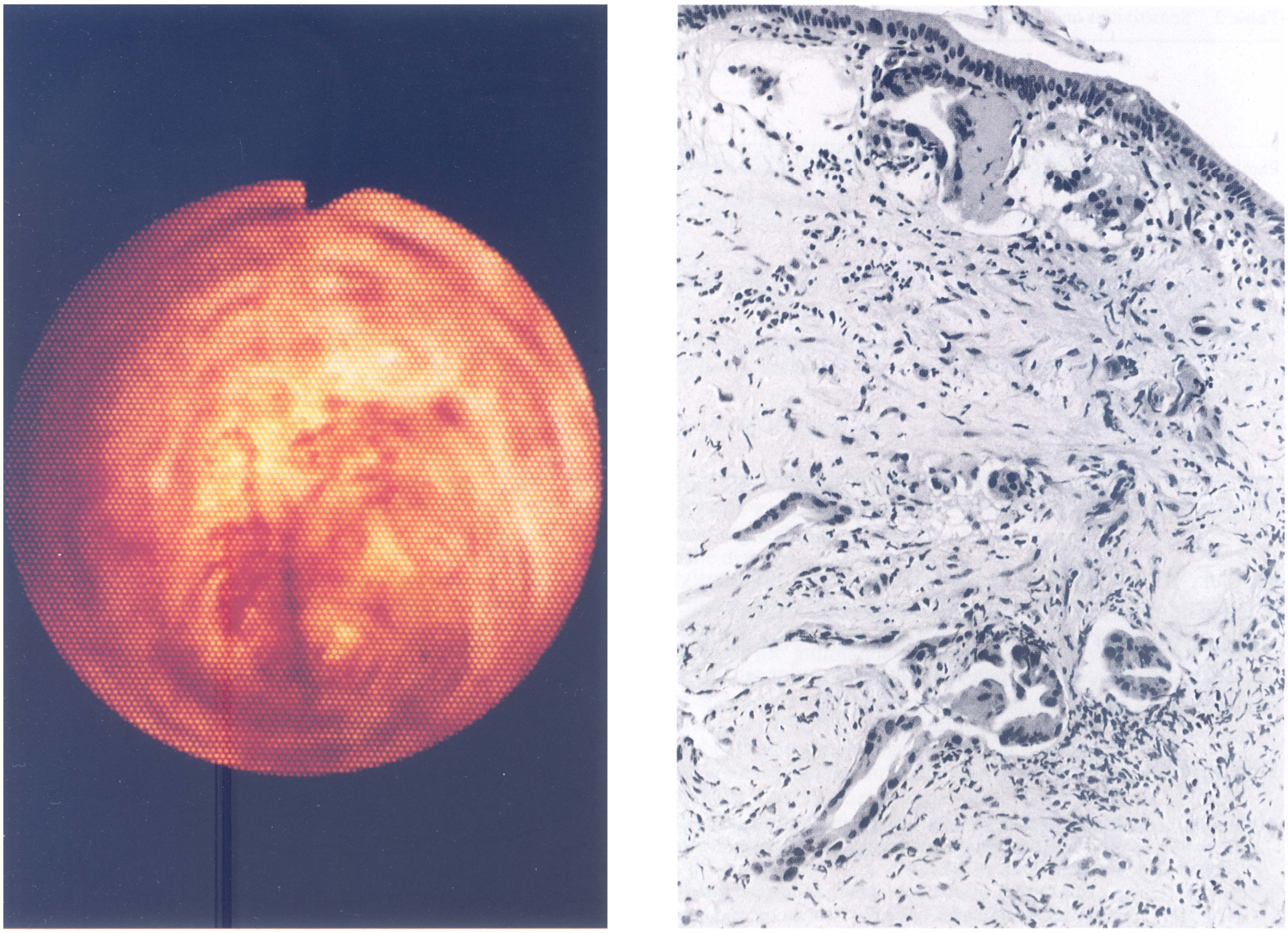

**Figure 3** A case of pancreatic carcinoma. PTCS feature (left). There was vascular dilation but no papillogranular surface. Histological feature of biopsy specimen (right). Carcinoma invaded the stroma of the bile duct wall. Proper epithelium covered the surface of the bile duct (H-E, x50).

papillary proliferation of superficial epithelia did not show the papillogranular surface and two regions associated with the papillary proliferation without noninvasive mucosal carcinoma showed papillogranular surface.

Vascular dilation is synonymous with tumor vessel (Kamiya *et al.*, 1988, 1989, Yamase *et al.*, 1988), capillary sign (Nakazawa *et al.*, 1985) and fine vascular proliferation (Nishikawa *et al.*, 1991), all reported previously. Nimura *et al.* (Nimura *et al.*, 1988) reported finding tumor vessels in all cases of bile duct carcinoma and carcinoma of the papillary region, and in 68% of the pancreatic carcinoma. Kamiya *et al.* (Kamiya *et al.* 1988) emphasized that this finding was characteristic of carcinomas that were exposed on the surface of the bile duct. However, in the present study, vascular dilation was related to invasive carcinoma, but not to exposure of the carcinoma on the surface of the bile duct. These findings resemble that observed by fiberoptic bronchoscopy in invasion of the bronchus by lung cancer. Amemiya *et al.* (Amemiya *et al.*, 1981) reported that vascular dilation was observed in the bronchial wall where adenocarcinoma of the lung invaded below normal epithelia of the bronchus. Still more, the high positivity for invasive carcinomas in biopsy materials taken from regions with vascular dilation suggests that vascular dilation indicated an invasion of carcinoma into shallow layers of the bile duct, because the biopsy specimen taken by forceps of 2 mm diameter were very small.

In addition, in regions without a papillogranular surface or vascular dilation, 50% (2/4) of biopsy materials taken from stenotic regions included carcinomas, while only 12% (2/17) of biopsy materials taken from nonstenotic regions included them. This indicates that a biopsy should be taken from stenotic regions regardless of existence of a papillogranular surface or vascular dilation.

In conclusion, our results indicate that a papillogranular surface and vascular dilation are characteristic of noninvasive mucosal carcinoma and invasive carcinoma, respectively. However, a papillogranular surface was related to the

**Table 3** Sensitivities and Specificities of PTCS Findings to Histologic Features

| | NIMCA | | INVCA | | PAP | |
|---|---|---|---|---|---|---|
| | (+) | (−) | (+) | (−) | (+) | (−) |
| Papillogranular surface | | | | | | |
| (+) (n=13) | 11 | 2 * | 7 | 6 | 12 | 1 * |
| (−) (n=44) | 3 | 41 | 22 | 22 | 0 | 44 |
| Sensitivity (%) | 79 | | | | 100 | |
| Specificity (%) | 95 | | | | 98 | |
| Vascular dilation | | | | | | |
| (+) (n=30) | 9 | 21 | 26 | 4 * | 8 | 22 |
| (−) (n=27) | 5 | 22 | 3 | 24 | 5 | 22 |
| Sensitivity (%) | | | 90 | | | |
| Specificity (%) | | | 86 | | | |

NIMCA: noninvasive mucosal carcinoma; INVCA: invasive carcinoma; PAP: papillary proliferation of superficial epithelia; *: P<0.0001, by chi-square test or Fisher's exact probability test.

papillary proliferation of superficial epithelia frequently noted in noninvasive mucosal carcinomas, rather than to cytological atypia of the epithelia. Further studies are needed to clarify differences between the papillogranular surfaces of carcinomas and of nonneoplastic epithelia.

### REFERENCES

Albores-Saavedra J., Henson D.E.: Tumor of the gallbladder and extrahepatic bile ducts. In Atlas of tumor pathology (2nd series), Armed Forces Institute of Pathology, 1986

Amemiya R., Oho K., Takizawa N., *et al.* Endoscopic findings and pathological findings of tracheobronchial disease. Evaluation of depth of invasion in the bronchial structure. *J Jap Bronchology* 1981;3:413–419 (in Japanese)

Kamiya J., Futamura Y., Hayakawa N., *et al.* Stereoscopic studies of vessels on the surface of bile duct carcinoma and bile duct mucosa involved by carcinoma. *Gastroenterol Endosc* 1988;30:337–345 (in Japanese)

Kamiya J., Nimura Y.: Diagnostic advantages of percutaneous transhepatic cholangioscopy (PTCS) on biliary and pancreatic carcinoma. *Bil Tract Pancreas* 1989;10:35–41 (in Japanese)

Nakazawa S., Naito Y., Kimoto E., *et al.* Utility of percutaneous transhepatic cholangioscopy (PTCS) in the diagnosis of bile duct cancer. *Bil Tract Pancreas* 1985;6:1339–1347 (in Japanese)

Nimura Y., Kamiya J., Hayakawa N., Shionoya S. Cholangioscopic differentiation of biliary strictures and polyps. *Endoscopy* 1989;21:351–356

Nishikawa K., Ogawa S., Sato M., *et al.* A study of malignant bile duct stenosis using percutaneous transhepatic cholangioscopy. *Dig Endosc* 1991;3:342–349

Sato M., Maetani I., Ogawa S., *et al.* Histopathological diagnosis of biopsy specimens of the bile duct. *Endosc Digest* 1992;4:1777–1782 (in Japanese)

Takada T., Suzuki S., Nakamura M., *et al.* Percutaneous transhepatic cholangioscopy as a new approach to the diagnosis of the biliary diseases. *Gastroenterol Endosc* 1974;16:106–111 (in Japanese)

Yamase H., Nimura Y., Hayakawa N., *et al.* Differential diagnosis on stenosis of distal bile duct by percutaneous transhepatic cholangioscopy (PTCS). *Gastroenterol Endosc* 1988;30:1175–1182 (in Japanese)

